# Knowledge of Back Pain and Spinal Disorders Among the General Population in the Western Region of Saudi Arabia

**DOI:** 10.7759/cureus.55587

**Published:** 2024-03-05

**Authors:** Raghad M Alsaqqa, Mohammed K Alghamdi, Khalid Basamih, Maria AlSulami, Maqbel Almajnooni, Ziyad S Al Saedi, Walaa H Alhudhudi, Mokhtar Shatla

**Affiliations:** 1 College of Medicine, Taif University, Taif, SAU; 2 Faculty of Medicine, Umm Al-Qura University, Makkah, SAU; 3 Faculty of Medicine, King Abdulaziz University, Rabigh, SAU; 4 Umm Alqura University, College of Medicine, Makkah, SAU

**Keywords:** western region, ksa:kingdom of saudi arabia, mechanical back pain, knowledge, inflammatory back pain, back pain

## Abstract

Introduction

Low back pain (LBP) is one of the most common global health problems and the second most common reason for seeking medical advice. However, most LBP does not indicate a serious disorder. Over half of the Saudi Arabian population experiences LBP at least once in their lives. Therefore, it is important to assess and understand how people manage this health problem. This study assessed back pain and spinal disorder knowledge among the general population in Saudi Arabia’s western region.

Methods

This was a cross-sectional study of the general population in western Saudi Arabia. The data were collected using an online, self-administered, Arabic version of the validated questionnaire about LBP. A statistical analysis of the collected data was performed using a software program.

Results

A total of 754 eligible participants completed the questionnaire. Less than half of the participants could correctly define acute and chronic LBP and sciatica. Only 19.2% of participants were aware that medical history and clinical examinations are used to diagnose LBP. Young participants, university graduates, and unmarried participants had good LBP knowledge.

Conclusion

This study showed that the general population of Saudi Arabia in the Western region needs more knowledge about the definitions of acute and chronic LBP. However, they had fair knowledge about the aggravating factors and triggers of LBP. Young participants had better knowledge about LBP. Awareness campaigns with brochures and flyers can be used to increase the population’s knowledge.

## Introduction

Low back pain (LBP) is one of the most prevalent health issues worldwide, the second most common reason for seeking medical advice [1], and a common leading cause of disability and absence from work. Additionally, LBP imposes a destructive economic burden on individuals and communities [2]. LBP is localized pain between the costal margin and inferior gluteal folds that may be accompanied by leg pain; however, in most cases, LBP does not indicate severe disorders [3,4]. Some studies indicate that nearly 8 in 10 people will experience LBP at least once in their lifetimes [2]. Generally, the onset of acute LBP symptoms occurs between ages 30 and 60, with the incidence peaking between 45 and 60 years of age. This condition is most common in people over 60, with a prevalence of 25.1% in males and 35.5% in females [5-7]. In a 2014 systematic review estimating the global prevalence of LBP, the total was 9.4% in 2010, with men more frequently affected than women (10.1% versus 8.8% prevalence, respectively) [1]. After adjusting for age, the same study found that LBP was more common in Western Europe than in the Middle East and central Latin America and less common in the Caribbean (15%, 14.8%, 6.6%, and 6.5%, respectively) [1].

A systematic review to determine the prevalence of LBP in Saudi Arabia found that the prevalence of LBP symptoms ranged from 53.2% to 79.1% [8]. Many factors can affect the human body’s musculoskeletal system and sometimes lead to LBP. Work-related activities such as lifting heavy objects and dealing with sharp objects, and individual factors such as advancing age, obesity, and stress, can cause the onset of or worsen these problems [9]. In Saudi Arabia, LBP is associated primarily with vitamin D deficiency and obesity. In addition, incorrect posture when carrying heavy objects, such as simultaneously lifting and twisting, as well as sudden torso movements, contribute to Saudi Arabia’s increased LBP incidence [10-13].

However, while LBP is not typically severe, it may be a sign of serious conditions, such as cancer, spinal fractures, infections, cauda equina syndrome, and aortic aneurysms [14]. Therefore, it was important for us to assess the general population’s level of knowledge to ensure that they have adequate knowledge of their disease and do not overestimate or underestimate their condition. Numerous studies have evaluated the same variable, but none have been conducted in Saudi Arabia’s Western region. Most of these studies concluded that the general population’s knowledge of the spine and its disorders needs to be increased [15].

## Materials and methods

This cross-sectional study targeted the entire population living in the western region of Saudi Arabia. In September 2023, the biomedical research ethics committee at Umm AlQura University approved the study (No. HAPO-02-K-012-2023-09-1720).

The study includes all adults, either male or female, Saudi or non-Saudi residents living in the Western region of Saudi Arabia. It excludes anyone with previous clinic visits for back pain, back or spine-related diseases, or psychiatric disorders. The sample size was estimated using the Sample Size Calculator program, with a confidence level of 95% and a margin of error of 5%. The data were collected via an online survey using Google Forms, which was shared with the general population on social media.

A total of 754 eligible participants completed the study questionnaire. The questionnaire was developed and validated by Maciel et al. [16] to assess the general population’s level of knowledge about LBP, including general aspects, concepts, and treatments. The questionnaire also included questions about basic anatomy, back pain, the definitions of various causes, diagnoses, and treatments. In addition, this questionnaire included demographic factors such as age, gender, monthly income, and education level. Electronic consent was obtained from each participant before they completed the questionnaire. A group of data collectors published the questionnaire online via social media platforms from September 2023 to December 2023.

The data were collected, reviewed, and fed into the Statistical Package for Social Sciences, version 21 (SPSS: An IBM Company, Armonk, NY). All statistical methods were two-tailed, with an alpha level of 0.05. The results were considered significant if the P-value was less than or equal to 0.05. According to Maciel et al. [16], the knowledge score was categorized as poor if the participants scored less than 60% of the overall score; if the participants scored 60% or more of the overall score, they were considered to possess a good level of awareness. Descriptive analysis was conducted by estimating the frequency distributions and percentages of the study variables. The participants’ knowledge of LBP was tabulated into different domains, and their overall knowledge was graphed. A cross-tabulation of the participants’ overall knowledge level distribution based on their personal data and other factors was conducted using the Pearson chi-square test for significance and the exact probability test for small frequency distributions.

## Results

A total of 754 eligible participants completed the study questionnaire. The participants’ ages ranged from 18 to more than 55 years, with a mean age of 26.7 ± 12.4 years. Among the participants, 501 (66.4%) were females, 560 (74.3%) had a university-level education or above, nearly half of the study participants (48.5%) were unmarried, and a small number (2.9%) were divorced (Table 1).

**Table 1:** Personal characteristics of the study population, Western region, Saudi Arabia

Personal data	No	%
Age in years		
18–25	321	42.6%
26–35	132	17.5%
36–45	153	20.3%
46–55	114	15.1%
>55	34	4.5%
Gender		
Male	253	33.6%
Female	501	66.4%
Educational level		
Below university (diploma and below)	194	25.7%
University/above (bachelor and above)	560	74.3%
Marital status		
Unmarried	366	48.5%
Married	366	48.5%
Divorced	22	2.9%

General knowledge of LBP among the population in the western region of Saudi Arabia (Table 2). A total of 36.5% of participants knew that the back and abdominal muscles supported the spine. Only 13% of the respondents defined LBP as pain between the lower thoracic ribs and the pelvis; 30.2% defined acute LBP as pain in the lower back area that usually disappears within three weeks, with or without treatment; and 42% defined chronic LBP as pain in the lower region of the back that lasts for more than three months. Moreover, 46.8% defined sciatic pain as pain between the lower thoracic ribs and the pelvis that extends to the legs and feet.

**Table 2:** General knowledge about low back pain among population in western region

General knowledge	No	%
For the general structure of the spine, choose one wrong answer		
The back and abdominal muscles do not help support the spine (expected answer)	275	36.5%
It consists of the cervical, thoracic, lumbar, and sacral vertebrae	99	13.1%
Between each vertebra and another, there is an intervertebral disc (disc) that absorbs shocks	106	14.1%
The vertebrae form a canal through which the spinal cord passes	67	8.9%
I don't know	207	27.5%
What is lower back pain?		
The pain is between the lower thoracic ribs and the pelvis (expected answer)	98	13.0%
The pain is between the lower thoracic ribs and the pelvis and extends to the legs and feet	147	19.5%
Pain in any area of the back, from the neck to the pelvis	244	32.4%
Pain in the abdomen, lower part of the pelvis, or kidneys	114	15.1%
I don't know	151	20.0%
What is acute lower back pain?		
It is pain in the lower back area and usually disappears within three weeks with or without treatment (expected answer)	228	30.2%
It is pain that cannot be treated in the lower region of the back	64	8.5%
It is pain in the lower back area that requires surgical intervention	112	14.9%
It is pain in the lower region of the back that lasts for more than three months	127	16.8%
I don't know	223	29.6%
What is chronic lower back pain?		
It is pain in the lower region of the back that lasts for more than three months (expected answer)	317	42.0%
It is pain in the lower back area and usually disappears within three weeks, with or without treatment	28	3.7%
It is pain that cannot be treated in the lower region of the back	98	13.0%
It is pain in the lower back area that requires surgical intervention	135	17.9%
I don't know	176	23.3%
What is sciatica?		
The pain is between the lower thoracic ribs and the pelvis and extends to the legs and feet (expected answer)	353	46.8%
The pain is between the lower thoracic ribs and the pelvis	24	3.2%
Pain in any area of the back, from the neck to the pelvis	91	12.1%
Pain in the abdomen, lower part of the pelvis, or kidneys	55	7.3%
I don't know	231	30.6%

Knowledge of the clinical features of LBP among the population in the western region of Saudi Arabia (Table 3). A total of 74.9% of respondents knew that pain in the lower area would worsen when carrying heavy objects, and 60.3% knew that difficulty in picking up things from below could be signs of LBP. In terms of causes, 81.2% knew about incorrect posture, arthritis, and herniated discs, but only 25.6% knew about tumors, infections, and fractures being causes of LBP. About 38.5% knew that medical history and clinical examination are used to diagnose LBP, and only 19.2% knew that X-rays are not needed.

**Table 3:** Public knowledge regarding low back pain clinical features, western region

Clinical features	No	%
Signs of lower back pain: choose two correct answers		
Pain in the lower area that gets worse when carrying heavy objects (expected answer)	565	74.9%
Fatigue and body pain	222	29.4%
Cough, lethargy, and loss of energy	34	4.5%
Difficulty in picking up things from below (expected answer)	455	60.3%
I don't know	101	13.4%
Causes of lower back pain, choose two correct answers		
Tumors, infections and fractures (expected answer)	193	25.6%
Diabetes mellitus	50	6.6%
Cold and old age	385	51.1%
Incorrect posture, arthritis and herniated disc (expected answer)	612	81.2%
I don't know	108	14.3%
Methods of diagnosis for lower back pain, choose two correct answers		
X-ray not needed (expected answer)	145	19.2%
Medical history and clinical examination (expected answer)	290	38.5%
Magnetic resonance imaging and computed tomography scan	461	61.1%
Lab investigations	128	17.0%
I don't know	189	25.1%

Knowledge of LBP treatment among the population in the western region of Saudi Arabia (Table 4). A total of 47.3% of participants knew that acute LBP requires one week of bed rest; only 21.5% knew that acute LBP requires the least amount of rest possible. 59.7% knew about exercises and instructions to protect the spine, which are used to treat chronic LBP. About 53.7% knew that surgical treatment may be required in some cases, and 36.7% knew that the majority of patients with acute LBP recover within three weeks.

**Table 4:** Public knowledge about treatment of low back pain, western region, Saudi Arabia

Treatment of low back pain	No	%
Treatment methods of acute lower back pain: choose two correct answers		
It requires the least amount of rest possible (expected answer)	161	21.5%
It requires one week of bed rest	355	47.3%
Requires sick leave from work	297	39.6%
Lower back pain may improve without treatment (expected answer)	189	25.2%
I don't know	205	27.3%
Treatment methods of chronic lower back pain: choose two correct answers		
Use a supportive belt when performing strenuous activities (expected answer)	406	53.8%
Physical means such as short waves, ultrasound	120	15.9%
Long-term use of anti-inflammatory medications	101	13.4%
Exercises and instructions to protect the spine (expected answer)	450	59.7%
I don't know	201	26.7%
Regarding surgical intervention to treat the lower back, choose two correct answers		
It is required in some cases.(expected answer)	405	53.7%
It is the best treatment for any type of lower back pain	105	13.9%
It may be important in cases of pressure on nerve roots and in cases of spinal cord dysfunction (expected answer)	408	54.1%
Surgical intervention ensures recovery from lower back pain	106	14.1%
I don't know	214	28.4%
For acute lower back pain, choose two correct answers		
The majority of patients recover within three weeks (expected answer)	277	36.7%
Awareness of how to protect the spine should be routine because the pain can recur (expected answer)	436	57.8%
After improving the pain, the patient will have completely recovered	117	15.5%
Instructions on how to protect the spine are only required when pain occurs	206	27.3%
I don't know	214	28.4%

The knowledge of the protective measures for spine and LBP among the population in the western region of Saudi Arabia (Table 5). In total, 33.3% of the respondents knew that intensive exercises are not required to treat acute LBP, 51.1% knew that wearing high heels all day is not good for the spine, and 63% knew that sitting while wearing shoes and socks is the best method to protect the spine.

**Table 5:** Public knowledge about protective measures for spine and low back pain, western region, Saudi Arabi

Protective measures	No	%
Regarding physical activities and lower back pain, choose one incorrect answer		
Intensive exercises are required to treat acute lower back pain (expected answer)	251	33.3%
Water activities may be useful in treating chronic lower back pain	85	11.3%
Walking three times a week for an hour can improve chronic lower back pain	107	14.2%
The most recommended exercises are exercises to strengthen the abdominal and lower back muscles	113	15.0%
I don't know	198	26.3%
Regarding spinal protection, choose one incorrect answer		
Wearing high heels all day (expected answer)	385	51.1%
You must get out of bed slowly, turning to the sides with the help of your hands	79	10.5%
Avoid carrying heavy objects on one side of the body (dividing the load on both sides)	112	14.9%
Avoid twisting the spine	46	6.1%
I don't know	132	17.5%
Methods to protect spine?		
Sitting when wearing socks and shoes (expected answer)	475	63.0%
The best way is to sleep on the stomach	166	22.0%
Picking up things from below without bending the knees	291	38.6%
Constant neck bending when reading	56	7.4%
I don't know	239	31.7%

**Figure 1:**
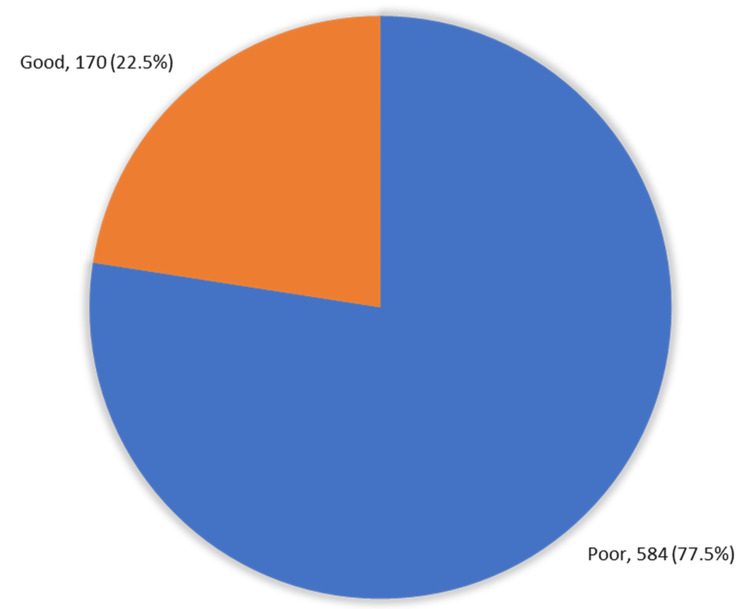
Overall knowledge about low back pain among study people, western region, Saudi Arabia

Only 170 (22.5%) participants demonstrated a good level of overall knowledge of LBP, while the majority (77.5%) had a poor level of knowledge (Figure 1).

Factors associated with the participants’ overall knowledge of LBP (Table 6). About 29.6% of young-aged participants demonstrated a good level of overall knowledge of LBP, compared with 15.8% of participants aged 46-55 years, with a recorded statistical significance of P = 0.001. Moreover, 24.1% of university graduates had a good knowledge level of LBP compared with 18% of the other participants (P = 0.048). Good knowledge of LBP was found among 30.1% and 9.1% of the unmarried and divorced participants, respectively (P = 0.001).

**Table 6:** Factors associated with participants' overall knowledge about low back pain P: Pearson X^2^ test; ^Exact probability test; P < 0.05 (significant).

Factors	Overall knowledge level				p-value
	Poor		Good		
	No	%	No	%	
Age in years					0.001*^
18–25	226	70.4%	95	29.6%	
26–35	103	78.0%	29	22.0%	
36–45	132	86.3%	21	13.7%	
46–55	96	84.2%	18	15.8%	
>55	27	79.4%	7	20.6%	
Gender					0.994
Male	196	77.5%	57	22.5%	
Female	388	77.4%	113	22.6%	
Educational level					0.048*
Below university (diploma and below)	159	82.0%	35	18.0%	
University/above (bachelor and above)	425	75.9%	135	24.1%	
Marital status					0.001*
Unmarried	256	69.9%	110	30.1%	
Married	308	84.2%	58	15.8%	
Divorced	20	90.9%	2	9.1%	

## Discussion

Low back pain is a prevalent health issue across all age groups and is regarded as a primary cause of disability [17-19]. The prevalence of LBP is estimated to be 10% at the age of 20 and 19.6% at ages 20-59 [20, 21]. LBP is more common in older individuals and affects 25.1% of men and 35.1% of women aged 60 years and older [22]. LBP has a prevalence of 64.6% and is a serious health issue in Gulf Cooperation Council nations [23-25].

Our study aimed to assess the knowledge of back pain and spinal disorders among the general population in the western region of Saudi Arabia. The study revealed that about 22% of the participants possessed a good knowledge of LBP. The results reported by Awwad et al. [26] and Tarimo et al. [27] show that, among the Saudi population, 8 out of 10 participants demonstrated similar awareness levels, with an overall median score of 9 (interquartile range: 0-19) out of 24 points. A total of 36.5% of our participants knew that the back and abdominal muscles helped support the spine. Only 13% correctly defined acute LBP, while a higher percentage correctly defined chronic LBP and sciatica. In terms of the clinical features of LBP, about 75% of the respondents knew that pain in the lower area worsens when carrying heavy objects.

Regarding the causes, about 81% of the respondents knew that incorrect posture, arthritis, and herniated discs could be causes of LBP. Only 25% of participants selected tumors, infections, and fractures, while half of them chose cold and old age. Nyagah and Merle [28] found that most patients living with LBP lack knowledge about its causes and contributing factors. Other studies have revealed that the general population, including patients diagnosed with LBP, has poor knowledge and awareness about the causes and contributing factors of LBP [29,30]. About the diagnosis of LBP, about 38% of our participants believe that medical history and clinical examination are used to diagnose LBP, and only 19% know that X-rays are unnecessary. On the other hand, about 61% of participants choose magnetic resonance imaging, and computed tomography scans are often used to diagnose LBP.

Poor knowledge of treatments for acute attack management was observed, but a much better awareness of chronic attack management was noted. Half of the study respondents were aware of the role of surgery as a management strategy. More than half of the participants knew that awareness of how to protect the spine should be routine because the pain can recur. Regarding the protective measures of the spine, 33% of the respondents knew that intensive exercises play no role in acute attacks; half of the participants knew that wearing high heels all day is not good for the spine; and 63% knew that the best method to protect the spine is sitting while wearing shoes and socks.

In terms of the factors associated with participants’ knowledge level about LBP, the current study reported that young participants, university graduates, and unmarried participants showed the significantly highest knowledge level. This was in concordance with other study findings on the effect of age on respondents' awareness levels [26]. A study in Germany revealed the need for more awareness of commonly available guidelines and an uneven distribution of existing knowledge throughout the population [30]. Furthermore, awareness campaigns through brochures and flyers can be used to raise the population’s knowledge about LBP and to plan health education programs for individuals with LBP.

Although our study had an adequate sample size compared to previous similar studies, it had some limitations in the method of collecting the data; it was collected through an online survey, which may lead to response bias. In addition, misunderstandings of the study questions could be happening; a phone or physical interview would be more effective.

## Conclusions

This study showed that the general population of the western region of Saudi Arabia had poor knowledge about low back pain, especially about the difference between acute and chronic LBP, the diagnosis, and the treatment. They had fair knowledge about its aggravating factors and triggers. Young participants, university graduates, and single participants demonstrated good knowledge about LBP. Awareness campaigns and brochures were recommended. 

## Appendices

Low back pain knowledge questionnaire:

Part one

Have you ever visited any health care provider due to back pain?

1- Yes

2- No

Have you ever been diagnosed with any disease affecting your back?

1- Yes

2- No

Have you ever been diagnosed with any mental illness?

1- Yes

2- No

Part two

Sex:

1- Male

2- Female

Age:

1- 18-25

2- 26-35

3- 36-45

4- 46-55

5- More than 55

Educational status:

1- University degree or higher

2- less than University degree

 
Social status:

1- Married

2- Divorced

3- Single

Income:

1- Less than 5000

2- 5,000-10,000

3- 10,000-15,000

4- 15,000-20,000

5- More than 20,000

Part Three

Please choose the required answer for each question. If you do not know the answer, please choose “I don’t know.”

 Regarding the general structure of the spine, choose one incorrect answer:

1- It consists of the cervical, thoracic, lumbar, and sacral vertebrae.

2- Between each vertebra there is an intervertebral disc that act as shock absorbs.

3- The vertebrae form a canal through which the spinal cord passes.

4- The back and abdominal muscles have no function in supporting the spinal column.

5- I don't know.

What is low back pain? Choose one correct answer:

1- The pain located between the lowest ribs and the pelvis.

2- The pain located between the lowest ribs and the pelvis that radiates down the leg to the foot.

3- Pain in any region of ​​the back, from the neck to the hip.

4- Pain in the abdomen, lower part of the pelvis, or kidneys.

5- I don't know.

What is acute low back pain? Choose one correct answer:

1- Pain located in the lumbar region that usually improved within three weeks, with or without treatment.

2- Untreatable pain in the lumbar region.

3- Pain in the lumbar region requiring surgery.

4- Pain in the lumbar region lasting more than three months.

5- I don't know.

What is chronic low back pain? Choose one correct answer:

1- Pain located in the lumbar region that usually improved within three weeks, with or without treatment.

2- Untreatable pain in the lumbar region.

3- Pain in the lumbar region requiring surgery.

4- Pain in the lumbar region lasting more than three months.

5- I don't know.

What is sciatica pain? Choose one correct answer:

1- The pain located between the lowest ribs and the pelvis.

2- The pain located between the lowest ribs and the pelvis that radiates down the leg to the foot.

3- Pain in any region of ​​the back, from the neck to the hip.

4- Pain in the abdomen, lower part of the pelvis, or kidneys.

5- I don't know.

These are symptoms of low back pain. Choose two correct answers:

1- Cough, lethargy, and loss of energy.

2- Fatigue and body pain.

3- Pain in the lumbar region that worsens when carrying heavy objects.

4- Difficulty in picking up objects from the floor.

5- I don't know.

These can cause low back pain. Choose two correct answers:

1- Cold and aging.

2- Postural problems, arthritis and a herniated disc.

3- Tumors, infections and fractures.

4- Diabetes mellitus. 

5- I don't know.

What is needed for the diagnosis of low back pain? Choose two correct answers:

1- MRI and CT scan are always needed for diagnosis.

2- X-ray is not always needed for diagnosis.

3- The diagnosis is often possible through the medical history and physical exam of the patient without the need of supplementary exams.

4- Laboratory tests such as glycemia, cholesterol and urine are always needed.

5- I don't know.

Regarding the treatment of acute lower back pain, choose two correct answers:

1- Requires one week of bed rest.

2- Requires sick leave from work.

3- Lowe back pain may improve without treatment.

4- The least possible rest is indicated.

5- I don't know.

What can be used to treat chronic low back pain? Choose two correct answers:

1- Long-term use of anti-inflammatory medications.

2- Instructions on spine protection and exercises.

3- Use a supportive belt when performing strenuous activities.

4- Physical means such as short waves, ultrasound, and Bier’s oven are more important than physical exercises.

5- I don't know.

Regarding physical activities and low back pain, choose one incorrect answer:

1- Walking three times a week for an hour can improve chronic low back pain.

2- Intensive exercises are indicated for acute low back pain.

3- Aquatic activities may be beneficial to the patient with chronic low back pain.

4- The most recommended exercises are strengthening of the abdomen and the back muscles, stretching and physical conditioning.

5- I don't know.

To protect the spine, choose two correct answers:

1- The best way to sleep is on the stomach.

2- Sit down when wearing socks and shoes.

3- Picking up objects from the floor without bending the knees.

4- Constant neck bending when reading.

5- I don't know.

Again, regarding protecting the spine, choose one wrong answer:

1- You should get out of bed carefully, turning sideways with the help of our hands.

2- Avoid carrying heavy objects on one side of the body (dividing the load on both sides).

3- Avoid twisting of the spine.

4- Wearing high heels all day.

5- I don't know.

About acute low back pain, choose two correct answers:

1- The majority of patients recover within three weeks.

2- After improving and recovering from the pain, the patient is cured and there is no risk for the pain to return.

3- Instructions on how to protect the spine are only important during the crisis.

4- Orientations for spine protection and energy conservation should be routine in patients with history of low back pain because relapses are frequent.

5- I don't know.

Regarding surgical intervention to treat the low back, choose two correct answers:

1- It is required in some cases.

2- It may be important in cases with nerve root compression and spinal column instability that do not improve with clinical treatment.

3- Surgery guarantees the cure of low back pain.

4- It is the best treatment for any type of low back pain.

5- I don't know.

Regarding drug treatment for low back pain, choose one wrong answer:

1- Anti-inflammatory medications and analgesics can be used in acute attacks.

2- Cortisone can be used in acute attacks.

3- Antidepressants and anti-epileptics can be used for chronic low back pain.

4- Topical treatments such as (gels, plasters, oils...) are always used.

5- I don't know.
